# Group-based trajectory analysis identifies varying diabetes-related cost trajectories among type 2 diabetes patients in Texas: an empirical study using commercial insurance

**DOI:** 10.1186/s12913-023-10118-1

**Published:** 2023-10-18

**Authors:** Gang Han, Matthew Scott Spencer, SangNam Ahn, Matthew Lee Smith, Lixian Zhong, Elena Andreyeva, Keri Carpenter, Samuel D. Towne, Veronica Averhart Preston, Marcia G. Ory

**Affiliations:** 1 Center for Community Health and Aging, Texas A&M University, College Station, TX United States of America; 2 Department of Epidemiology and Biostatistics, School of Public Health, Texas A&M University, College Station, TX United States of America; 3 Department of Health Management and Policy, College for Public Health and Social Justice, Saint Louis University, St. Louis, MO United States of America; 4 Department of Health Behavior, School of Public Health, Texas A&M University, College Station, TX United States of America; 5 College of Pharmacy, Texas A&M University, College Station, TX United States of America; 6 Department of Health Policy and Management, School of Public Health, Texas A&M University, College Station, TX United States of America; 7 School of Global Health Management and Informatics, University of Central Florida, Orlando, FL United States of America; 8 Disability, Aging, and Technology Cluster, University of Central Florida, Orlando, FL United States of America; 9 Southwest Rural Health Research Center, Texas A&M University, College Station, TX United States of America; 10 Blue Cross and Blue Shield of Texas a subsidiary of Health Care Service Corporation, Richardson, TX USA; 11 Department of Environmental and Occupational Health, School of Public Health, Texas A&M University, College Station, TX United States of America

**Keywords:** Type 2 Diabetes related cost, Group-based trajectory analysis, Multivariable logistic regression models, Diabetes self-management education and support

## Abstract

**Background:**

The trend of Type 2 diabetes-related costs over 4 years could be classified into different groups. Patient demographics, clinical factors (e.g., A1C, short- and long-term complications), and rurality could be associated with different trends of cost. Study objectives are to: (1) understand the trajectories of cost in different groups; (2) investigate the relationship between cost and key factors in each cost trajectory group; and (3) assess significant factors associated with different cost trajectories.

**Methods:**

Commercial claims data in Texas from 2016 to 2019 were provided by a large commercial insurer and were analyzed using group-based trajectory analysis, longitudinal analysis of cost, and logistic regression analyses of different trends of cost.

**Results:**

Five groups of distinct trends of Type 2 diabetes-related cost were identified. Close to 20% of patients had an increasing cost trend over the 4 years. High A1C values, diabetes complications, and other comorbidities were significantly associated with higher Type 2 diabetes costs and higher chances of increasing trend over time. Rurality was significantly associated with higher chances of increasing trend over time.

**Conclusions:**

Group-based trajectory analysis revealed distinct patient groups with increased cost and stable cost at low, medium, and high levels in the 4-year period. The significant associations found between the trend of cost and A1C, complications, and rurality have important policy and program implications for potentially improving health outcomes and constraining healthcare costs.

## Introduction

Over 37 million Americans had diabetes in 2021, with 90–95% having Type 2 diabetes (T2D) [1]. Additionally, T2D diagnoses have been occurring at younger ages and higher rates than previous decades [1]. Diabetes’ national prevalence has more than doubled in the last 20 years due to an increase in obesity and growing aging population, although there is some indication that new diabetes cases may be plateauing due, in part, to better diabetes awareness and prevention [2]. In 2017, a total of $327 billion was attributed to diabetes-related medical costs and lost work in the United States, and the healthcare expenditures of adults living with diabetes ($16,752) are 2.3 times higher than those without diabetes ($7,151) from the same population [3]. Furthermore, diabetes-related complications account for 48-64% of medical expenses for patients with T2D [4].

In Texas, diabetes prevalence has consistently surpassed the national average from 1999 to 2019. In 2019, 12.9% of Texans (2.7 million people) had diabetes, while 10.9% of all Americans had diabetes [5]. In 2017, the annual cost of diabetes and prediabetes in Texas was estimated to be $25.6 billion in diabetes-related costs [6]. The growing economic burden of diabetes makes it critically important to understand factors related to health care utilization and costs and how to best target interventions that help individuals effectively prevent and manage diabetes.

A variety of risk factors are associated with the presence of diabetes, including a lack of physical activity, obesity, high blood pressure, family history of diabetes, and residing in rural areas [7]. Additional risk factors include short- and long-term complications such as hypoglycemia, heart disease, retinopathy, neuropathy, and chronic kidney disease [8]. A1C values can provide critical insight into the average blood sugar level of patients for the past 2–3 months, with higher values corresponding to greater risk of developing diabetes-related complications [9].

To quantify the magnitude of increased medical costs for patients with diabetes, several studies have investigated the relationship between A1C values and diabetes-related costs. For example, Bansal et al. [10] and Smith et al. [11] identified a statistically significant positive relationship between A1C levels and diabetes-related costs. Lage and Boye [12] found that a 1% reduction in A1C was on average associated with a 6.9% reduction in diabetes-related healthcare costs, which is equivalent to $555 in annual cost savings. Candrilli and colleagues [13] established the relationship between A1C reduction and costs of an episode of care for diabetic patients who experience certain comorbidities, such as coronary artery disease and cerebrovascular disease.

Statistical methods used in the above literature provided unique insights into the relationship between diabetes risk factors and the corresponding medical costs, which can facilitate future expenditure estimates among key stakeholders and policymakers. These findings also highlighted the importance of controlling A1C values to alleviate escalating diabetes-related healthcare costs. However, there is a potential flaw in interpreting findings from prior studies. Although traditional statistical models can identify key factors associated with T2D related cost with high statistical power given large sample sizes, such analyses ignore the fact that patients are likely to be highly heterogeneous. A statistical analysis based on the entire sample could miss important signals in subsets of the T2D population. For example, at any A1C level, some patients may have increased cost over time, while others may have relatively stable costs at low and high levels. The heterogeneity and other features in the dataset, including excessive zeros and outliers, can result in a high amount of “noise” in the observations, which can hinder a statistical model’s ability to identify clinically significant factors associated with different trajectories of cost.

In the context of these methodological difficulties, this article investigates the use of group-based trajectory modeling [14, 15] for modeling the relationship between T2D-related cost and T2D risk factors using longitudinal data from 2016 to 2019 commercial claims in Texas. Jones et al. [14] and Jones and Nagin [15] developed the group-based trajectory models, a type of mixture models for estimating developmental trajectories, and implemented them in a procedure named “Proc Traj” in the statistical analysis software (SAS). This analytical tool can provide a mechanism to classify different patterns of change over time. As such, the follow-up analysis based on different patterns has the clear advantage of reducing majority of the “noise” and outliers in the cost observations. We define patients having the same pattern of cost changed over time to be in the same cluster. By creating and analyzing these clusters, we aim to provide novel insights regarding the pattern of changes in cost for patients with T2D. It is worthwhile to note the statistical analysis software “Proc Traj” has been utilized to analyze projected costs. For example, Karampampa et al. [16] investigated cost trajectories among newly diagnosed multiple sclerosis cases. Four trajectories were identified to describe clusters of high or low direct and indirect costs associated with multiple sclerosis. The trajectories suggested that illness costs may be associated with severity of disease and the use of disease-modifying therapies. As another example, Lauffenburger et al. [17] used “Proc Traj” to classify changes in spending patterns by Medicare beneficiaries into five distinct trajectories. The ability to accurately group these distinct changes into spending patterns helped identify and implement targeted interventions, which could reduce the medical spending from Medicare beneficiaries.

However to our knowledge, “Proc Traj” has not been used to cluster cost trajectories for patients with diabetes, and has not been used to correlate the clusters (or groups) with key factors including A1C, complications, and metropolitan residence status of the patients. To fill this research gap, this study aims to achieve three objectives: (1) to understand the trajectories of cost in different groups; (2) to investigate the relationship between cost and key factors in each cost trajectory group; and (3) to assess significant factors associated with different cost trajectories.

## Methods

### Study sample

We used 2016–2019 commercial claims data in Texas provided by a large commercial insurer. Individuals with type 2 diabetes were identified using the *International Classification of Diseases*, Tenth Revision (ICD10), code E11 [18], and hemoglobin A1C values between 4 and 14 were considered valid to account for variability in the sensitivity of measurement instruments across clinical settings. This analysis targeted individuals who were: (1) commercially insured; (2) Texas residents; (3) diagnosed with T2D; and (4) ages 18–64 years since total costs for older adult enrollees could not be fully determined. Finally, participants were excluded if they had three or less T2D-related cost records over the four-year study period to ensure each participant had sufficient observations of cost to indicate a trend and to achieve reasonable classification of the trends in our analysis. The “cost” in this study is referred to as the actual paid amount by the patient and/or insurance.

### Human subjects research designation

Given the utilization of de-identified secondary administrative claims records, the Texas A&M Institutional Board ( IRB # IRB2020-0204) determined that this was not human subjects research, and did not require informed consent. Since all data was de-identified, it was not possible to get individual informed consent. See our declaration of ethics approval and consent to participate for further information.

### Patient flow

**Fig. 1:**
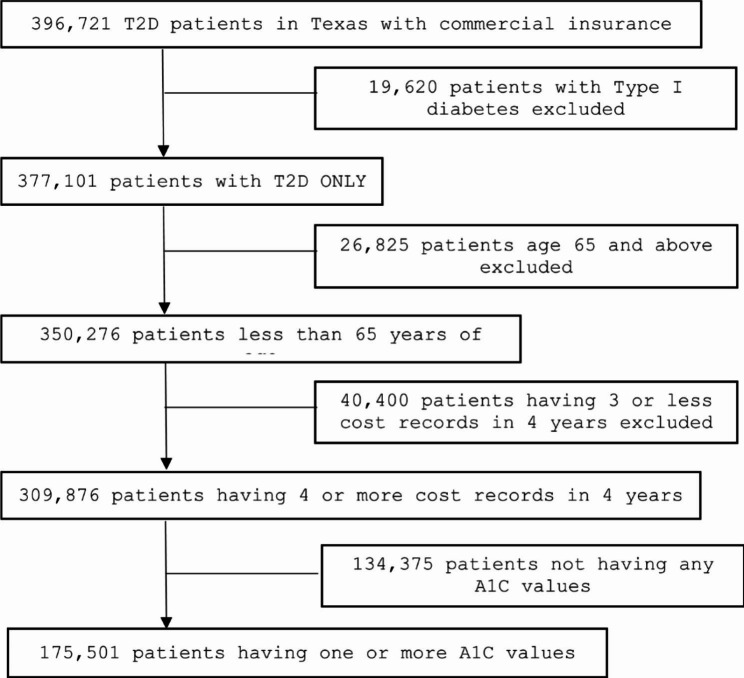
Patient flow diagram

Figure 1 illustrates a total of 309,876 patients were included in our analysis of the T2D related cost after the exclusions of Type 1 diabetes, age greater or equal to 65, and those who had less than 4 quarterly health records during the four years period. Note that analyses including A1C values were reduced to 175,501 patients (Table 1). Additionally, this is not a true cohort study with four-year records on everyone, but rather an analysis of patients with the designated number of cost records over a four-year study period as the original dataset did not include data on the initial cohort for four years. Data was obtained on all individuals who had a diabetes diagnosis, regardless of when the diagnosis occurred. For instance, individuals who were diagnosed in 2016 were included in the dataset starting for that year, while individuals diagnosed in 2018 were included in the dataset starting in that year. To create a simulated cohort, the study focused on individuals who had at least four cost records any time over a four-year period. These cost records could be from the same or different years.

**Table 1:** Descriptive statistics for all patients and patients in each group. Median, interquartile range [IQR] and sample size are reported for each continuous variable; frequency and percentage (%) are reported for each categorical variable

Variable	Level	All	Group 1	Group 2	Group 3	Group 4	Group 5	P-value
A1C baseline		6.9, [6.1, 8.4] N = 175,501	6.3, [5.8, 7.7] N = 16,064	6.8, [6.1, 8.5] N = 10,541	6, [5.5, 6.7] N = 28,495	7, [6.3, 8.6] N = 58,384	7.4, [6.5, 8.9] N = 62,017	< 0.0001
A1C category	1, A1C < 5.7	20,564 (12%)	3064 (19%)	1161 (11%)	9938 (35%)	4147 (7%)	2254 (4%)	< 0.0001
	2, 5.7 < = A1C < 6.5	46,487 (26%)	5715 (36%)	3275 (31%)	10,064 (35%)	15,327 (26%)	12,106 (20%)	
	3, 6.5 < = A1C < 8	60,309 (34%)	4083 (25%)	3607 (34%)	4564 (16%)	21,409 (37%)	26,646 (43%)	
	4, 8 < = A1C	48,141 (27%)	3202 (20%)	2498 (24%)	3929 (14%)	17,501 (30%)	21,011 (34%)	
Each patient median A1C		6.9, [6.1, 8.2] N = 175,501	6.4, [5.8, 7.4] N = 16,064	6.7, [6.1, 7.9] N = 10,541	5.9, [5.5, 6.7] N = 28,495	7, [6.2, 8.4] N = 58,384	7.3, [6.5, 8.5] N = 62,017	< 0.0001
Age		53, [45, 58] N = 175,501	49, [41, 56] N = 16,064	51, [43, 57] N = 10,541	50, [41, 57] N = 28,495	53, [46, 58] N = 58,384	55, [48, 59] N = 62,017	< 0.0001
Sex	Male	94,113 (54%)	8621 (54%)	5749 (55%)	14,313 (50%)	32,901 (56%)	32,529 (52%)	< 0.0001
	Female	81,376 (46%)	7442 (46%)	4791 (45%)	14,181 (50%)	25,479 (44%)	29,483 (48%)	
	Other	12 (0%)	1 (0%)	1 (0%)	1 (0%)	4 (0%)	5 (0%)	
Complication	1, No	74,589 (43%)	9344 (58%)	4751 (45%)	19,733 (69%)	25,862 (44%)	14,899 (24%)	< 0.0001
	2, Short only	49,489 (28%)	4141 (26%)	3105 (29%)	5516 (19%)	17,712 (30%)	19,015 (31%)	
	3, Long only	25,811 (15%)	1918 (12%)	1495 (14%)	2764 (10%)	8495 (15%)	11,139 (18%)	
	4, Both	25,608 (15%)	661 (4%)	1188 (11%)	481 (2%)	6314 (11%)	16,964 (27%)	
Metro Status	1, metro	155,721 (89%)	14,325 (89%)	9169 (87%)	25,427 (89%)	51,648 (88%)	55,152 (89%)	< 0.0001
	2, non-metro	19,780 (11%)	1739 (11%)	1372 (13%)	3068 (11%)	6736 (12%)	6865 (11%)	
Continuous CCI*		1, [1, 2] N = 175,078	1, [1, 2] N = 15,877	1, [1, 2] N = 10,450	1, [1, 1] N = 28,403	1, [1, 2] N = 58,336	2, [1, 3] N = 62,012	< 0.0001
Categorical CCI	1,<=2	134,937 (77%)	14,346 (89%)	8577 (81%)	26,423 (93%)	48,775 (84%)	36,816 (59%)	< 0.0001
	2, >2	40,564 (23%)	1718 (11%)	1964 (19%)	2072 (7%)	9609 (16%)	25,201 (41%)	

*CCI: Charlson Comorbidity Index*

### Measurements and variables

Among the variables in the commercial claims data, we focused on the following measurements in our analysis.

- *Hemoglobin A1C.* The glycated hemoglobin (A1C) test measures the average plasma glucose in the previous eight to 12 weeks [19]. Assessment was limited to those who had A1C values in their claims record. A1C was measured in terms of the mean and median values at baseline as defined as the first measurement in the data set. When observing A1C longitudinally, A1C is quarterly measured value.
- *Sociodemographic characteristics*. Patients’ baseline age (18 to 64) and sex (male/female) were used in analyses. Race/ethnicity was not systematically recorded in the data and was not included in the analyses.
- *Non-metropolitan or metropolitan status*. Our measure of rurality was based on the patient’s county of residence and ZIP code from a database maintained by the National Center for Health Statistics (NCHS), the NCHS Urban-Rural Classification Scheme for Counties [20]. The NCHS Urban-Rural Classification Scheme has 6 levels. Among the 6 levels, 4 levels are for metropolitan areas, including large central metro, large fringe metro, medium metro, small metro; two levels are for non-metropolitan areas including micropolitan, non-core. In our analysis, we used a binary variable according to the above 4 and 2 levels to distinguish between metropolitan and non-metropolitan areas. We employ the term metro status to reflect associations with metro and non-metro geographic residence.
- *Diabetes-Related Complications*. Indications of short- and long-term diabetes-related complications were included in analyses. Short-term diabetes-related complications included hyperglycemia, hypoglycemia with coma, hyperosmolarity with coma, and hyperosmolarity without nonketotic hyperglycemic-hyperosmolar coma. Long-term diabetes-related complications included nephropathy, chronic kidney disease, retinopathy, neuropathy, foot ulcers, and skin ulcers. The presence of any short- and/or long-term complications over the four-year period were scored as two binary variables and combined to create a single 4-category variable scored 0 (no complications), 1 (only short-term complications), 2 (only long-term compilations), and 3 (both short- and long-term complications).
- *Charlson Comorbidity Index (CCI)*. The CCI values were available for patients based on the ICD10 diagnosis codes [18]. In each quarter the CCI scores are continuous ranging from 0 (no comorbidities) to 17 with a higher CCI value corresponding to a more severe comorbidity. In our analyses we will be treating CCI as a continuous variable, and based on highest CCI value over the four years.

### Study outcome

The study outcome is the T2D-related cost for paid claims. Annual and quarterly costs were computed by adjusting the total annual days available in the data to calculate costs per patient, which was used in group-based trajectory analysis. The costs refer to the total amount paid by the insurer and the patient to the provider. These are not charges but actual claims paid. This is the total money the provider receives for services rendered.

### Statistical analysis

#### Setting groups using trajectory of cost

To discover different trends of T2D-related cost over 4 years, group-based trajectory analyses [14, 15] were performed after logarithmic transformations of the cost (natural log of “cost + 1” so that 0 cost remains 0 in the log scale) to normalize the right-skewed distributions. The group-based method used only the cost to set groups without involving other measurements. The goal of fitting a group-based trajectory model was to identify for different cost trajectories, where each trajectory was for individuals following similar progressions of cost.

In this article we use the term “*groups*” to represent distinct trajectories of cost over time. The group-based trajectory analysis assumed each group has its own trajectory of cost measured over time, and treated the observed cost as a function of time. Each group potentially had its own patient characteristics and conditions for A1C and other complications, which may not be identified in an analysis of all patients. The group-based trajectory analysis identified discrete groups by maximizing the observed data likelihood of Type 2 diabetes-related cost, where the likelihood is the probability or chance of observing the data under the assumed model structure and parameter values.

The assumed model in our analysis of log cost is censored normal (model CNORM in SAS procedure “Traj”) left censored at 0. The time in quarter was treated as a polynomial covariate with linear, quadratic, and cubic terms. As proposed in Jones et al. [14], Bayesian information criteria (BIC) was used to decide the optimal number of groups with the smallest BIC value. In this example, we chose the total number of groups to be 2, 3, 4, 5, or 6 to start running the program. The fitted model with 5 groups had the smallest BIC value. In the classification program (SAS procedure “Traj”), each patient was classified into a group based on the highest posterior density. Specifically, suppose there are G groups (labelled as 1 to G). For patient “i” the observed cost values are data **Y**_i_ at times **T**_i_. The posterior density of patient “i” in group “g” is proportional to the product of (1) likelihood of data (**Y**_i_, **T**_i_) in group “g” and (2) prior density of group “g”. This classification procedure can be conducted in SAS software, procedure “Traj”. Classifying patients into different clusters and analyzing data from clusters can reveal additional insights into the change of cost and associated factors [15]. By separating cost trajectories into different groups, we can better capture the trend of cost by regulating “noise” in the data from excessive zeros, outliers, and random variations.

#### Descriptive statistics based on the groups

The aforementioned measurements and outcome were summarized numerically. Descriptive statistics, including median and interquartile range (IQR) for continuous variables as well as frequency and percentage for categorical variables were provided for all patients and patients in each of the 5 groups set by the group-based trajectory analysis. Continuous variables were compared using Kruskal–Wallis one-way analysis of variance, and discrete variables were compared using Pearson’s Chi-square test.

#### Within and between group modeling

The estimated trajectories of cost in the 5 groups (Fig. 1) indicated that groups 1 and 2 had low cost initially and increased later, group 3 had stable low cost, group 4 had stable medium cost, and group 5 had stable high cost. It is worthwhile to note groups 1 and 2 both had an increasing trend. The difference in the trajectories from groups 1 and 2 was mainly due to different enrollment times of participants in the commercial insurance. Despite different trajectories, groups 1 and 2 had essentially the same trend. Therefore, we reserved the term “*clusters*” to denote distinct patterns or trends of the cost over time. The 5 groups (trajectories) in Fig. 1 indicated 4 distinct clusters (trends of cost), which are increasing (groups 1 and 2), stable low (group 3), stable medium (group 4), and stable high (group 5).

We investigated the effect of the aforementioned 4 clusters in two ways. The first way (within group analysis) was to model the relationship between cost and measurements in each group. Generalized linear longitudinal regression was implemented to model the log of quarterly cost and longitudinal measures of A1C, adjusting the observation time in quarter, age, sex, rurality, short- and long-term complications, and CCI. Observations from the same individual was adjusted as cluster effects in SAS software procedure GENMOD. Polynomial regression terms of A1C, including the linear, quadratic terms, and the interaction between A1C and time were tested in the model to account for possible non-linear relationship between A1C and cost.

The second way (between group analysis) was to model whether and how some of the cluster levels were associated with the measurements, where two or more cluster levels were a categorical response variable, and other measurements (age, sex, complications, and CCI) were independent variables. Specifically, if the response included the two clusters of “increasing trend” and “stable low trend,” we can select significant variables associated with a higher possibility of increased cost over time. By modeling three clusters “stable low trend,” “stable medium trend,” and “stable high trend,” we can identify significant variables associated with the relatively stable cost. Logistic regression and ordinal logistic regression, with the reference level in the outcome variable being “stable low trend,” were employed to assess the predictors of trends of cost. Significant predictors were identified using univariable and multivariable logistic regression models with backward variable selection, and odds ratio (OR) estimates, 95% confidence intervals (CI), and p-values were reported.

In all the analyses, p-values less than or equal to 0.05 were considered statistically significant. All analyses were conducted using SAS software version 9.4 (SAS, NC, USA).

## Results

### Group-based trajectory analysis of type 2 Diabetes related cost over 4 years

**Fig. 2:**
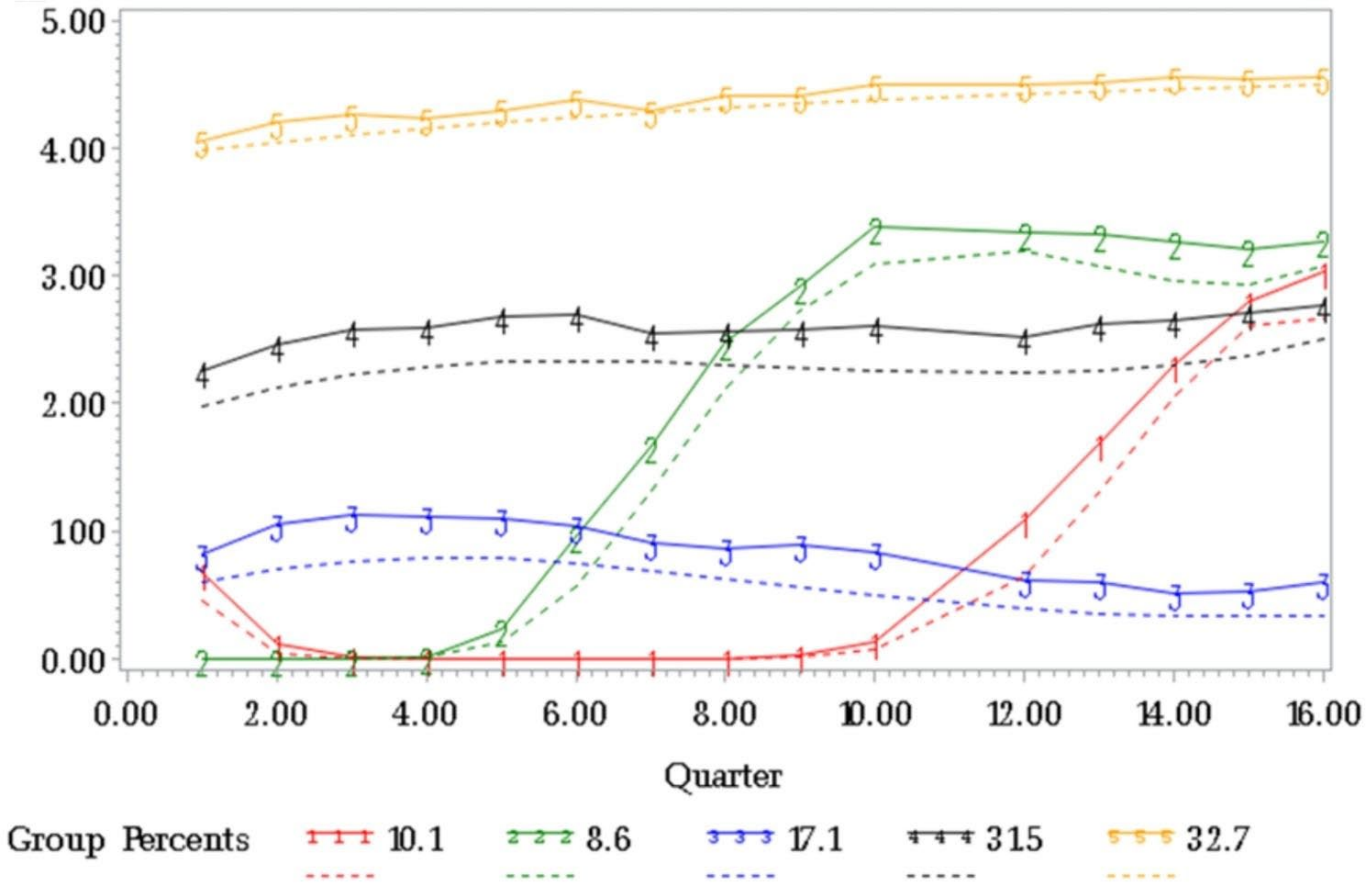
Group-based trajectory analysis fitting 5 group of log cost with different trends

The group-based trajectory analysis results are shown in Fig. 2. Groups 1 and 2, with 10.1% and 8.6% patients had increasing cost trends, respectively. The cost in Group 1 increased at 10–15 quarters, and the cost in Group 2 increased at 5–10 quarters. Groups 3, 4, and 5 had relatively low, medium, and high stable cost, with 17.1%, 31.5%, and 32.7% of patients, respectively.

### Descriptive statistics

Table 1 shows descriptive statistics for all patients and patients within each of the 5 groups. The median age was around 50 years each for Groups 1–3, but was 53 in Group 4, and 55 in Group 5. Around 55% of patients were female and 45% were male. The baseline, median A1C (over 16 quarters) was the lowest for the low stable cost group (i.e., A1C = 6), was higher for patients having increased cost (i.e., A1C ranging from 6.3 to 6.8), and was even higher for the medium and high cost groups (i.e., A1C ranging from 7.0 to 7.4). The proportions of A1C > = 8% was 30% in group 4, and 34% in group 5, which were higher than those in Groups 1–3. Compared with the stable low cost group (group 3), a significantly higher proportion of short- and long-term complications were observed in the medium and high cost groups, as well as the increasing cost group. For example, only 2% patients had both short- and long-term complications in the stable low cost group, but 4-10%, 10%, and 25% patients had short- and long-term complications with increasing cost trend, stable medium cost, and stable high cost, respectively. In all 5 groups, about 13–16% of patients were in non-metro areas. The stable low cost group had lowest CCI (7% observations with CCI > 2), and the stable high cost group had highest CCI (40% observations with CCI > 2). Based on the statistics in Table 1, higher T2D-related cost was correlated with older age, higher A1C, more complications, and higher CCI. Compared with stable low cost, increasing cost trend was correlated with more complications and higher CCI.

### Within group modeling

As illustrated in Table 2, we estimated and tested the key factors in a linear longitudinal regression model for cost (log scale) in the 4-year time period, where time was in terms of quarters and ranged from 1 to 16. We fit the model for all patients, patients having increasing cost trend (groups 1 and 2 combined), patients having stable low cost (group 3), medium cost (group 4), and high cost (group 5). Although statistically significant (primarily due to the large sample size), the effect of age was small or nearly negligible in all subgroups. For the low-cost group, male sex was associated with the high cost. While in the high cost group, female sex was associated with high cost. In all subgroups, A1C was positively correlated with cost, but the association was higher in the stable low cost group (estimate 0.164, 95% CI: 0.155–0.173) and increasing cost group (estimate 0.133, 95% CI: 0.126–0.141) than the medium and high cost groups (estimates 0.067 and 0.043, respectively). As an interpretation of these estimates, one unit of increase of A1C was, on average, corresponded to 17.8% increase of cost in the stable low cost group, and 14.2% increase in the increasing cost group, but only 7% and 4% increase in the medium and high cost groups, respectively. In all subgroups, short- and long-term complications were significantly associated with higher cost.

**Table 2:** Multivariable longitudinal analysis of quarterly reported T2D-related cost over 4 years

Variable	Parameter estimate	95% Confidence Interval	P-value
**All patients**			
Age (continuous)	-0.001	-0.002– -0.000	0.0004
Sex (female vs. male)	0.018	0.009–0.027	< 0.0001
A1C (continuous)	0.093	0.090–0.095	< 0.0001
Complication (short vs. none)	0.325	0.315–0.334	< 0.0001
Complication (long vs. none)	0.292	0.276–0.307	< 0.0001
Complication (Both short and long vs. none)	0.852	0.823–0.881	< 0.0001
Metro status (non-metro vs. metro)	-0.053	-0.067– -0.039	< 0.0001
Charlson Comorbidity Index (Continuous)	0.209	0.201–0.218	< 0.0001
**Patients with increasing cost trend**			
Age (continuous)	-0.004	-0.006– -0.003	< 0.0001
Sex (female vs. male)	-0.019	-0.050–0.011	0.2111
A1C (continuous)	0.133	0.126–0.141	< 0.0001
Complication (short vs. none)	0.502	0.473–0.531	< 0.0001
Complication (long vs. none)	0.461	0.412–0.510	< 0.0001
Complication (Both short and long vs. none)	1.254	1.1422–1.366	< 0.0001
Metro status (non-metro vs. metro)	-0.025	-0.069–0.019	0.2666
Charlson Comorbidity Index (Continuous)	0.082	0.054–0.110	< 0.0001
**Patients with low stable cost trend**			
Age (continuous)	-0.011	-0.012– -0.009	< 0.0001
Sex (female vs. male)	-0.093	-0.128– -0.058	< 0.0001
A1C (continuous)	0.164	0.155–0.173	< 0.0001
Complication (short vs. none)	0.519	0.485–0.554	< 0.0001
Complication (long vs. none)	0.543	0.482–0.605	< 0.0001
Complication (Both short and long vs. none)	1.442	1.252–1.632	< 0.0001
Metro status (non-metro vs. metro)	0.060	0.009–0.111	0.0216
Charlson Comorbidity Index (Continuous)	-0.109	-0.136– -0.081	< 0.0001
**Patients with medium stable cost trend**			
Age (continuous)	-0.005	-0.005– -0.004	< 0.0001
Sex (female vs. male)	0.000	-0.013–0.014	0.9602
A1C (continuous)	0.067	0.0633–0.070	< 0.0001
Complication (short vs. none)	0.220	0.206–0.234	< 0.0001
Complication (long vs. none)	0.169	0.145–0.193	< 0.0001
Complication (Both short and long vs. none)	0.734	0.683–0.786	< 0.0001
Metro status (non-metro vs. metro)	-0.081	-0.101– -0.061	< 0.0001
Charlson Comorbidity Index (Continuous)	0.112	0.098–0.127	< 0.0001
**Patients with high stable cost trend**			
Age (continuous)	-0.006	-0.006– -0.005	< 0.0001
Sex (female vs. male)	0.038	0.026–0.050	< 0.0001
A1C (continuous)	0.043	0.040–0.047	< 0.0001
Complication (short vs. none)	0.189	0.177–0.201	< 0.0001
Complication (long vs. none)	0.129	0.109–0.149	< 0.0001
Complication (Both short and long vs. none)	0.594	0.559–0.628	< 0.0001
Metro status (non-metro vs. metro)	-0.056	-0.076– -0.036	< 0.0001
Charlson Comorbidity Index (Continuous)	0.265	0.256–0.275	< 0.0001

Comparing the significant associations across the different groups, the association between geographic residence (i.e., metro vs. non-metro) and cost varied between groups. With increasing cost trend, metro status was not significantly associated with cost (p = 0.266). For stable low-cost group, patients with non-metro status had higher cost than those with metro status (estimate 0.060, 95% CI 0.009–0.111). For medium and high cost groups, patients with non-metro status had lower cost than those with metro status, respectively.

Higher CCI was significantly positively correlated with cost in the high cost group (estimate 0.265, 95% CI 0.256–0.275). On average, one unit increase of CCI corresponded to 30% increase in the T2D-related cost in the high cost group.

### Between group modeling

The between group analysis had two parts. Part 1 identified factors associated with increasing cost trend versus stable low cost, and Part 2 identified factors associated with the magnitude of cost. For Part 1, we fit univariable and multivariable logistic regression models to test the effects of age, sex, baseline A1C, complication, metro status, and CCI. The outcome was binary with 2 levels of low stable cost (Group 3 in Fig. 2) and increased cost (Groups 1 and 2 in Fig. 2) over the 4 years. Results are summarized in Table 3. Older age was significantly associated with the increased cost over time. Sex was not significantly associated with the increasing trend after adjusting other variables. Baseline A1C was significantly associated with the increasing trend in both univariable and multivariable analysis with ORs 1.237 and 1.188, respectively. Both short- and long-term complications were associated with the increasing trend. The level of having both short- and long-term complications had the highest likelihood of increasing cost trend, with ORs 4.608 and 3.545 in the univariable and multivariable analyses, respectively. Patients in non-metro areas had significantly higher likelihood of increasing cost trend compared with those in metro areas, where the ORs were 1.110, 95% CI (1.072, 1.151) in the univariable and 1.105, 95% CI (1.046, 1.167) in the multivariable analysis. CCI was positively associated with cost over time, where the ORs were 1.953, 95% CI (1.872, 2.038) in the univariable and 1.133, 95% CI (1.113, 1.154) in the multivariable analysis for continuous CCI.

**Table 3:** Univariable and multivariable logistic regression analyses of low stable cost vs. increasing cost over 4 years. (N = 98,973)

Variable	Odds Ratio	95% Confidence Interval	P-value
Univariable analysis			
Age (continuous)	1.006	1.005–1.008	< 0.0001
Sex (female vs. male)	0.886	0.864–0.908	< 0.0001
Baseline A1C (continuous)	1.237	1.225–1.249	< 0.0001
Complication (short vs. none)	1.691	1.640–1.744	< 0.0001
Complication (long vs. none)	1.565	1.504–1.629	< 0.0001
Complication (Both short and long vs. none)	4.608	4.267–4.976	< 0.0001
Metro status (non-metro vs. metro)	1.110	1.072–1.151	< 0.0001
Charlson Comorbidity Index (continuous)	1.203	1.187–1.218	< 0.0001
Multivariable analysis			
Age (continuous)	1.002	1.000–1.003	0.0397
Sex (female vs. male)	0.976	0.942–1.011	0.1717
Baseline A1C (continuous)	1.188	1.176–1.200	< 0.0001
Complication (short vs. none)	1.562	1.496–1.631	< 0.0001
Complication (long vs. none)	1.497	1.414–1.584	< 0.0001
Complication (Both short and long vs. none)	3.545	3.185–3.944	< 0.0001
Metro status (non-metro vs. metro)	1.105	1.046–1.167	0.0004
Charlson Comorbidity Index (continuous)	1.133	1.113–1.154	< 0.0001

*Low stable cost is the reference level*

In the analysis of Part 2, identification of factors associated with the magnitude of cost, we fit univariable and multivariable cumulative logistic regression models to compare stable high cost, stable medium cost, and stable low cost subgroups, treating low cost as reference. The outcome had 3 ordinal levels of low stable cost (Group 3 in Fig. 2), medium stable cost (Group 4 in Fig. 2), and high stable cost (Group 5 in Fig. 2) over the 4 years. Results are shown in Table 4 where age, baseline A1C, short- and long-term complications, and CCI are associated with higher amount of cost. Although not associated with increasing trend (p = 0.172, Table 3, multivariable analysis), female sex was significantly associated with higher cost in Table 4, with OR 1.049, 95% CI (1.034, 1.064) in univariable analysis, and OR 1.103, 95% CI (1.081, 1.126) in multivariable analysis. While non-metro area had significantly higher likelihood of the increasing trend than metro area (p = 0.0004 in Table 3, multivariable analysis), metro status was not significant in the multivariable analysis for stable costs after adjusting other factors (p = 0.198 in Table 4).

**Table 4:** Univariable and multivariable cumulative logistic regression analyses of low stable vs. medium stable vs. high stable cost over 4 years. Low stable cost is the reference level. (N = 260,984)

Variable	Odds Ratio	95% Confidence Interval	P-value
Univariable analysis			
Age (continuous)	1.040	1.039–1.041	< 0.0001
Sex (female vs. male)	1.049	1.034–1.064	< 0.0001
Baseline A1C (continuous)	1.260	1.253–1.267	< 0.0001
Complication (short vs. none)	2.502	2.457–2.547	< 0.0001
Complication (long vs. none)	3.018	2.953–3.085	< 0.0001
Complication (Both short and long vs. none)	8.021	7.821–8.227	< 0.0001
Metro status (non-metro vs. metro)	0.978	0.958–0.999	0.036
Charlson Comorbidity Index (Continuous)	1.668	1.656–1.680	< 0.0001
Charlson Comorbidity Index (> 2 vs. < = 2)	4.441	4.358–4.525	< 0.0001
Multivariable analysis			
Age (continuous)	1.040	1.039–1.041	< 0.0001
Sex (female vs. male)	1.103	1.081–1.126	< 0.0001
Baseline A1C (continuous)	1.178	1.171–1.185	< 0.0001
Complication (short vs. none)	2.461	2.401–2.524	< 0.0001
Complication (long vs. none)	2.084	2.953–3.085	< 0.0001
Complication (Both short and long vs. none)	4.646	4.479–4.819	< 0.0001
Metro status (non-metro vs. metro)	1.021	0.989–1.054	0.198
Charlson Comorbidity Index (Continuous)	1.372	1.358–1.385	< 0.0001

*Low stable cost is the reference level*

## Discussion

To better understand the trend of Type 2 diabetes-related cost paid by commercial insurance, we implemented the group-based trajectory analysis [14, 15] and identified significant factors associated with different trend patterns. The group-based trajectory analysis showed in Fig. 2 revealed distinct patterns in the trajectories of the T2D-related cost over the 4-year study period. The group-based trajectory methods are flexible to capture different trends and not restricted to monotone changes [14, 15]. We identified groups of patients having either stable cost or the increasing trend in relation to diabetes and its complications. The identification and controlling of the significant factors could provide insights into healthcare utilization, healthcare resources planning, and future policy recommendations.

With the clusters from the group-based trajectory analysis (Fig. 1), we provided descriptive statistics (Table 1) and within group (Table 2) and between group (Tables 3 and 4) analyses to test the effects of multiple measurements. Compared with the analysis for the entire sample (Table 2, all patients), our analysis revealed additional insights. Of note, Table 3 examines factors associated with higher possibilities of having increasing cost in the future 1–4 years given that the current Type 2 diabetes-related cost is relatively low. The group-based trajectory analysis ensures the objectivity and not-by-chance when classifying participants with increasing cost.

Our study expands upon existing studies that examine rising costs of diabetes care over time [3] or identify key clinical, sociodemographic and economic factors associated with poorer diabetes management and higher costs [21]. Interestingly, having employer-based insurance does not negate the costs of care for persons with diabetes with out-of-pocket costs still being a major patient concern [22–24]. Consistent with prior reviews revealing an association between diabetes and accelerated aging processes [25], older age was associated with the increasing trend over time and higher stable cost over time. Explicating prior research on sex differences associated with health care expenditures [26], male sex was associated with higher possibility of having increasing cost, while female sex was associated with higher cost if the cost remained stable. Consistent with other studies showing a relationship between A1C and costs [11], for the entire patient sample and all subgroups, higher A1C was associated with higher cost and higher possibility of the increasing trend over the 4 years. Similarly, confirming other studies [4] complications and CCI were generally associated with higher cost and higher possibility of the increasing trend.

Adding nuances to previous studies demonstrating high prevalence of diabetes and related mortality in rural areas [27–30], patients in non-metro areas had slightly higher cost in some of the groups (i.e., patients with stable and relatively low cost), but they had lower cost for the entire patient group relative to patients in metro areas. In our analysis, we discovered the non-metro status is associated with higher possibility of the increasing trend, but had little or non-significant association with higher cost given that the cost was stable over the 4 years.

We were especially interested in examining rural-urban differentials given the plethora of data indicating a disproportionate burden of T2D [31], poor access to preventive health services [32], higher diabetes mortality rates in rural areas [33]. There have been suggestions that lower costs in rural areas might reflect a health disparity difference in access to care [34] with slightly lower diabetes screening rates for those living in rural areas [30]. Such health disparities in diabetes self-management can result in significant downstream health care burdens and costs [35, 36].

The group-based trajectory analysis had methodological advantages over other approaches such as classification and regression trees and distance-based methods (such as k-means clustering and nearest neighbor methods). First, the group-based trajectory analysis builds clusters based on trends of cost over time, and covariates are not involved in the process of clustering [15]. Second, this approach can identify significant factors associated with, not only the magnitude of cost, but also the trend of cost over time, which is valuable for understanding the change of cost and factors associated with the change. Specifically, as shown in Tables 3 and 4, this approach enabled us to differentiate between: (a) factors associated with a higher likelihood of increasing cost trend over time; and (b) factors associated with higher cost amount if cost was relatively stable over time. Third, this method can accommodate missing values in the longitudinal outcome measurements given that some patients had missing cost values up to 12 out of 16 quarters over the 4 years in this commercial claims data. Fourth, although the censored normal distribution was used on the log scale, this method is able to assume other distributions for different data types, for example, binary, ordinal, and count variables [15].

Despite its promising results, this study has some important limitations that should be acknowledged. A noted limitation relates to the group-trajectory analysis is that a group with a low sample size may be selected only by chance, which can limit the generalizability for groups representing small proportions (for example, 1–3%) of the overall sample size. Given our total sample size was large (close to 200,000 patients with A1C values), and the proportion of each of the 5 groups are relatively high in Fig. 1 (at least 8.6%), the five groups were likely representative of different cost trajectories. Another possible shortcoming of this study lies in the patient inclusion of commercially insured Texas residents. These findings could apply to other Texas residents with commercial insurance, but may not apply to residents outside of Texas, patients with non-commercial insurance (e.g., Medicare and Medicaid), or patients without any medical insurance.

Other limitations can be noted that are typical of secondary data of existing large administrative insurance claims data bases. While we were able to include A1C in our analyses as a major clinical factor, we acknowledge that lab values such as A1C are not routinely included in claims data [37] and appears to be a reporting issue. In fact, A1Cs were only reported for slightly more than half of the claims data records. While we can document rural-urban cost differentials, we are unable to provide definitive explanatory factors for the rural-urban differences that emerged in our analyses. The existing dataset did not contain information about “major procedures” related to diabetes critical illness. Further, the dataset only contains to total costs per quarter, not the number of the number of claims per year. Additionally, since working rural residents are less likely to be covered by private insurance given employment in jobs that do not provide employer insurance coverage, there may be a selection bias in our sample. We do note, though, that the data base includes individuals getting their insurance through health exchanges set up by the affordable care act [38].

Given these limitations within the data, we are unable to speculate, for example, about the nature of the stable high cost groups among rural-dwellers or other rural-urban differences. Finally, it is worthwhile to note the data set for this work does not have information regarding insulin usage, metformin, glucose monitoring, and certain complications such as end stage kidney diseases. This is because less than half of commercial members in our sample had pharmacy coverage with the same insurer as their medical coverage. The rest were covered by other pharmacy benefit managers so that their pharmacy claims were carved out from the claims. Future work should focus on the role and impact of different therapeutic regimens on the patients’ diabetes control and management as well as types of diabetes-related costs (e.g., outpatient visits, inpatient care, regular clinical visits) and associated trends. Investigation of cost type could be informative, for example, hospitalization costs may be more expensive than outpatient visits due to medications, lab tests, consultation and patient education programs. Linking survival information with cost would be insightful. Clinic visits and length of stay information can be useful to explain the rural/urban disparity.

The findings of our analysis have important policy and program implications. Regular A1C monitoring is still important for managing diabetes and preventing its complications (e.g., heart disease, chronic kidney disease, nerve damage) [39]. As such, implementing Diabetes Self-management Education and Support (DSMES) in communities can improve patient’s diabetes self-management, their glucose control, and their satisfaction with treatment and care received [40]. The Diabetes Education Program, a form of DSMES, observed a significant reduction of A1C among study participants in the South Texas region and estimated healthcare cost savings up to $5.6 million over a two-to-three-year period [11, 41]. Our current study suggests significant associations of A1C and complications with the T2D-related cost (Tables 2 and 4) and the change of cost over time (Table 3). With a higher A1C and more complications at baseline, the low cost at baseline is more likely to increase over time. As a result, managing diabetes complications via diabetes self-management programs should be the first-line therapeutic behavioral choice among patients with diabetes. Enhancing the enrollment of patients with high A1C, complications, but relatively low T2D related cost in self-management programs could be helpful to improve their health outcomes and reduce the potential healthcare costs.

## Conclusion

Group-based trajectory analysis revealed distinct patient groups with increasing cost trend and stable cost at low, medium, and high levels in the 4-year study period. Key factors of age, sex, A1C, short- and long-term complications, CCI, and metro status were included in the models for cost in each group, for the two groups of increasing versus stable low cost, and for the three groups of low, medium, and high stable cost. A1C and complications were significantly associated with T2D-related cost and the increasing trend of cost over the 4-year period. Living in rural areas was significantly associated with the increasing trend. These findings have important policy and program implications on healthcare costs and health outcomes in terms of targeting those at high risk for diabetes prevention programming and ensuring equitable access to care.

## Data Availability

The data that support the findings of this study are available from Blue Cross and Blue Shield of Texas (BCBSTX) but restrictions apply to the availability of these data, which were used under license for the current study, and so are not publicly available. Data are however available from the authors upon reasonable request and with permission of Blue Cross and Blue Shield of Texas. We refer all data access inquiries to the BCBSTX point of contact for this collaborative effort (Mark Chassey, Chief Medical Officer. Mark_Chassay@bcbstx.com) or the Texas A&M point of contact for all data (Mr. Jim Colson, Texas A&M Vice President, Digital Health, jim.colson@tamu.edu).

## Abbreviations

*T2D*: Type 2 diabetes
*A1C*: Glycated hemoglobin
*SAS*: Statistical Analysis System
*ICD10*: International Classification of Disease, Tenth Revision
*NCHS*: National Center for Health Statistics
*CCI*: Charlson Comorbidity Index
*BIC*: Bayesian information criteria
*IQR*: Interquartile range
*OR*: Odds ratio
*CI*: Confidence intervals
*DSMES*: Diabetes Self-management Education and Support
